# Spatiotemporal Pattern of PM_2.5_ Concentrations in Mainland China and Analysis of Its Influencing Factors using Geographically Weighted Regression

**DOI:** 10.1038/srep40607

**Published:** 2017-01-12

**Authors:** Jieqiong Luo, Peijun Du, Alim Samat, Junshi Xia, Meiqin Che, Zhaohui Xue

**Affiliations:** 1 Key Laboratory for Satellite Mapping Technology and Applications of State Administration of Surveying, Mapping and Geoinformation of China, Nanjing University, Nanjing 210023, China; 2 Jiangsu Provincial Key Laboratory of Geographic Information Science and Technology, Nanjing University, Nanjing 210023, China; 3 Jiangsu Center for Collaborative Innovation in Geographical Information Resource Development and Application, Nanjing 210023, China; 4 State Key Laboratory of Desert and Oasis Ecology, Xinjiang Institute of Ecology and Geography, Chinese Academy of Sciences, Urumqi 830011, China; 5 Research Center for Advanced Science and Technology, The University of Tokyo 4-6-1 Komaba, Meguro-ku, Tokyo 153-8904, Japan; 6 School of Earth Sciences and Engineering, Hohai University, Nanjing 211100, China

## Abstract

Based on annual average PM_2.5_ gridded dataset, this study first analyzed the spatiotemporal pattern of PM_2.5_ across Mainland China during 1998–2012. Then facilitated with meteorological site data, land cover data, population and Gross Domestic Product (GDP) data, etc., the contributions of latent geographic factors, including socioeconomic factors (e.g., road, agriculture, population, industry) and natural geographical factors (e.g., topography, climate, vegetation) to PM_2.5_ were explored through Geographically Weighted Regression (GWR) model. The results revealed that PM_2.5_ concentrations increased while the spatial pattern remained stable, and the proportion of areas with PM_2.5_ concentrations greater than 35 μg/m^3^ significantly increased from 23.08% to 29.89%. Moreover, road, agriculture, population and vegetation showed the most significant impacts on PM_2.5_. Additionally, the Moran’s I for the residuals of GWR was 0.025 (not significant at a 0.01 level), indicating that the GWR model was properly specified. The local coefficient estimates of GDP in some cities were negative, suggesting the existence of the inverted-U shaped Environmental Kuznets Curve (EKC) for PM_2.5_ in Mainland China. The effects of each latent factor on PM_2.5_ in various regions were different. Therefore, regional measures and strategies for controlling PM_2.5_ should be formulated in terms of the local impacts of specific factors.

Clean air is regarded as a basic requirement of human comfort, health and well-being. As we all know, air pollution has many serious adverse effects, such as negative impacts on the climate, ecosystem services, biodiversity, and food security^1^ and continuously posing severe threats to human health worldwide. According to a study of World Health Organization (WHO), more than two million people prematurely died each year attribute to the effects of air pollution since the 21st century. It is worth noting that more than half of these deaths happened in developing countries, particularly in China and India^2^. Particulates, especially PM_2.5_ (aerosol particles with aerodynamic diameter less than 2.5 μm), are the deadliest form of air pollutants. Epidemiologic studies have shown that exposures to PM_2.5_ are associated with increased cardiovascular and respiratory morbidity and mortality^3^. With the rapid development of urbanization and industrialization, PM_2.5_ pollutions in China, especially in the North part, is becoming serious, which can be clearly observed by satellite measurements^4^,^5^,^6^. Thus, the analysis of the spatiotemporal pattern of PM_2.5_ across China is very imperative for carrying out environmental epidemiologic studies.

For the past couple of years, most of the studies have investigated the spatiotemporal pattern of PM_2.5_ in China on a regional scale^7^,^8^,^9^,^10^. For instance, Liu *et al*.^7^ found that there were no significant differences of PM_2.5_ concentrations between urbanized and suburban areas of Shanghai. Meanwhile, from 2006 to 2008, the seasonal variation of PM_2.5_ in Hangzhou was noticeably characterized by higher concentration in winter and lower in summer^8^, which is consistent with the results of Xi’an^9^ and Beijing cities^10^. In recent years, the exploration of spatial and temporal variability of PM_2.5_ pollutions in these areas has expanded to more broad regional^11^,^12^ and national scales^13^,^14^. As aforementioned, most studies of the spatiotemporal pattern of PM_2.5_ at a regional scale are conducted with a short time scale and high temporal resolution, whereas those on a large regional or national scales usually use inter-annual and long time series data sets with a lower spatial resolution (e.g., 50 km). There has been a lack of research on the spatiotemporal pattern of PM_2.5_ concentrations in China with high spatial resolution using long time series data. Therefore, one of the primary objectives of our study is to analyze the spatiotemporal pattern of PM_2.5_ concentrations in China using more than a decade of data.

From the microcosmic perspective, a considerable number of existing studies have been dedicated to investigating the main sources of PM_2.5_ in China. They mainly focused on one large city at a specific time spot. For example, Liu *et al*.^15^ investigated the source apportionment of PM_2.5_ using observations in Beijing, a highly polluted city in Northern China. Wang *et al*.^16^ found that industrial process and vehicle emission are the dominant local contributors to total PM_2.5_ mass in the whole city. Also, analysis of the source apportionment of PM_2.5_ in the Yangtze River Delta of China indicated that PM_2.5_ is primarily from secondary pollutants and primary emissions from vehicles and biomass burning^17^.

From the macroscopic perspective, many previous studies have evaluated the impacts of natural geographical factors on PM_2.5_ concentrations at a regional scale, including vegetation^18^,^19^,^20^,^21^,^22^ and meteorological parameters^23^,^24^,^25^. However, limited attention was paid to explore the relationships between PM_2.5_ and socioeconomic factors. For instance, Lin *et al*.^13^ found that there was a significant positive correlation between population growth, economic development, urban expansion and PM_2.5_ concentrations. Other studies also identified that urbanization indicators (i.e., urban built-up area, population and industry fraction) have great impacts on urban PM_2.5_ concentrations^26^,^27^. In addition, Han *et al*.^28^ found the evidence of the increasing effects of human activities on PM_2.5_ pollutions through analyzing the relationship between artificial surface, cropland and PM_2.5_ concentrations.

Unfortunately, to the best of our knowledge, there is no research to systematically and comprehensively analyze the impacts of natural geographical and socioeconomic factors on PM_2.5_ at a national scale. Given the fact that China is facing severe air pollution and PM_2.5_ is its main component, to investigate the relationships between PM_2.5_ concentrations and its influencing factors is very important and valuable for drafting appropriate air pollution control policies. Thus, considering the fact that PM_2.5_ concentrations vary over space and there probably exists spatial autocorrelation within PM_2.5_ concentrations of surrounding regions, another primary objective of our study was to explore the contributions of the influencing factors, including natural geographical and socioeconomic factors on PM_2.5_ in 343 cities of Mainland China using geographically weighted regression (GWR) model. It is necessary for China in achieving the goals of sustainable development and the National New-type Urbanization Plan^28^,^29^.

In brief, this study has three main contributions. First, using more than a decade of data, we analyzed the spatiotemporal patterns of PM_2.5_ concentrations in China at a high spatial resolution. Second, to our knowledge, this research was the first attempt to investigate the relationships between PM_2.5_ concentrations and the natural geographical and socioeconomic factors across Mainland China on a city level from the macroscopic perspective. Last but not least, GWR, a local form of linear regression was applied in this study to fully consider the spatial heterogeneity of PM_2.5_. And it is of paramount importance for formulating and refining local pollution control strategies.

## Results and Discussion

### Spatiotemporal patterns of PM_2.5_ concentrations in Mainland China

As Supplementary Fig. S1 shown, in general, PM_2.5_ concentrations increased over most of Mainland China and its spatial pattern remained stable during the study period, and there were two main clusters of regions with low PM_2.5_ concentrations. One is located at the northern Inner Mongolian Plateau, and northeastern plains. The other is lied in the southwestern Tibetan Plateau. On the contrary, there are three main clusters of regions with high PM_2.5_ concentrations, including the North China plains, Yangtze plains and Central China, followed by Tarim Basin, and the Sichuan Basin. Many studies have partly attributed the higher PM_2.5_ concentrations in these regions to the coal-based industries such as coal-fired power plants, iron and steel manufacturing^17^,^30^. Comparatively, partly thanks to the developed tertiary industry which produces little pollution, the PM_2.5_ concentrations in the Pearl River Delta are the lowest in the three main economic zones^27^. The spatial distribution areas of PM_2.5_ concentrations greater than 100 μg/m^3^ during the two periods of 2005–2007 and 2006–2008 were larger than in other periods. During the period of 1998–2012, areas that exceeded Interim Target-1 35 μg/m^3^ expanded from the central eastern region to south western region, and eventually connected in the Sichuan Basin. In the southwestern Tibetan Plateau, northern Inner Mongolian Plateau, and northeastern plains, PM_2.5_ concentrations are lower than the WHO air quality guideline (AQG) of 10 μg/m^3^. Besides, PM_2.5_ concentrations of the southwestern Tibetan Plateau changed very little, while those of the northern Inner Mongolian Plateau and northeastern plains significantly increased. We can also observe that the tendency of high PM_2.5_ concentrations was still remaining high with a little decreasing trend.

**Figure 1:**
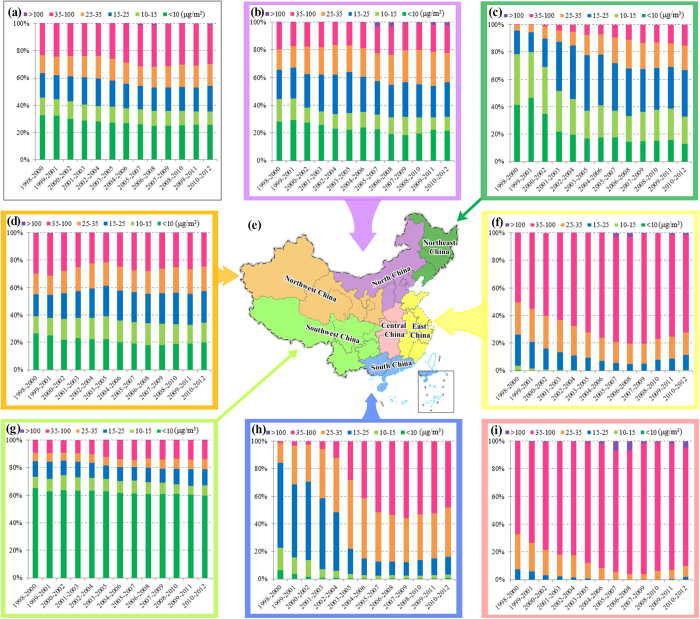
Annual variations of different grades of annual average PM_2.5_ concentrations in whole Mainland China and seven geographical subareas from 1998 to 2012. (**a**) Mainland China, (**b**) North China, (**c**) Northeast China, (**d**) Northwest China, (**e**) Subarea Map, (**f**) East China, (**g**) Southwest China, (**h**) South China, (**i**) Central China. The subarea map was generated in ArcGIS10.2, URL: http://www.esrichina-bj.cn/softwareproduct/ArcGIS/.

Furthermore, in order to quantitate the annual variations of all grades of PM_2.5_ concentrations in the whole Mainland China and seven geographical subareas from 1998 to 2012, the cumulative proportion of area of each concentration range was calculated as shown in Fig. 1.

The proportion of area experiencing low PM_2.5_ concentrations of Grade 1 significantly decreased from 32.92% to 25.67% and this decrease mostly occurred in Northeast, North and South China, slightly in Southwest and Northwest China. However, the proportion of area under high PM_2.5_ concentrations increased annually during the study period. For example, the proportion of area in Grade 6 increased from 0.27% to 0.91% and this increase primarily happened in East, North and Central China, while the proportion of area in Grade 5 significantly increased from 22.81% to 28.98%. Similarly, the proportion of area under mid-range concentrations also increased, with Grades 3 and 4 respectively increasing from 17.93% to 18.76% and from 13.32% to 15.88%, respectively, while Grade 2 decreased from 12.75% to 9.79%, mostly locating in South, Northeast and North China. The increase of Grade 3 was mainly located in Northeast, North and Northwest China, whereas declined in South, East and Central China. Meanwhile, the increase of Grade 4 came from South, North, Northeast and Northwest China, while declined in Central and East China.

The proportion of area whose PM_2.5_ concentrations is greater than the secondary standard of 35 μg/m^3^ increased from 23.08% to 29.89%. Likewise, the areal extent of PM_2.5_ concentrations between the primary and secondary standard increased from 31.25% to 34.64%, which is slightly more than one-third of the study area. Among the concentration grades, Grade 5 (35–100 μg/m^3^) shows the biggest area of increasing distribution, and this increase occurred primarily in East, South and Central China, slightly in Northeast China. The inter-annual undulation in area proportion was the largest for comprising Grades 3 and Grade 4, with both of these areas first rapidly decreasing and then slightly increasing. The largest area proportion of Grade 6 with PM_2.5_ concentrations greater than 100 μg/m^3^ happened at the periods 2005–2007 and 2006–2008. The largest area proportion under Grade 3 with PM_2.5_ concentrations of 15–25 μg/m^3^ occurred during 2002–2004, with an area ratio of 20.35%, then declined to 17.00% during 2006–2008, and finally increased to 18.76% during 2010–2012.

From Fig. S1 and Fig. 1, it should be stressed that three-fourths of Mainland China had PM_2.5_ concentrations that exceeded the WHO AQG of 10 μg/m^3^. The concentrations were particularly high in Central, North, Southwest and East China, and the most severe areas were the northern Henan, southern Hebei and Shanxi, central Shaanxi, eastern Sichuan and western Shandong. It is worth noticed that there was a rapid increase of highest PM_2.5_ concentrations (>100 μg/m^3^) over southern Hebei, northern Henan and western Shandong during 1998–2008, with peaks during 2007–2009, followed by a significant reduction during 2008–2010. It is believed that this reduction was associated with the national energy conservation policies that were carried out during the “Eleventh Five-Year” period^10^.

### Relationship between PM_2.5_ concentrations and latent geographic factors

**Figure 2:**
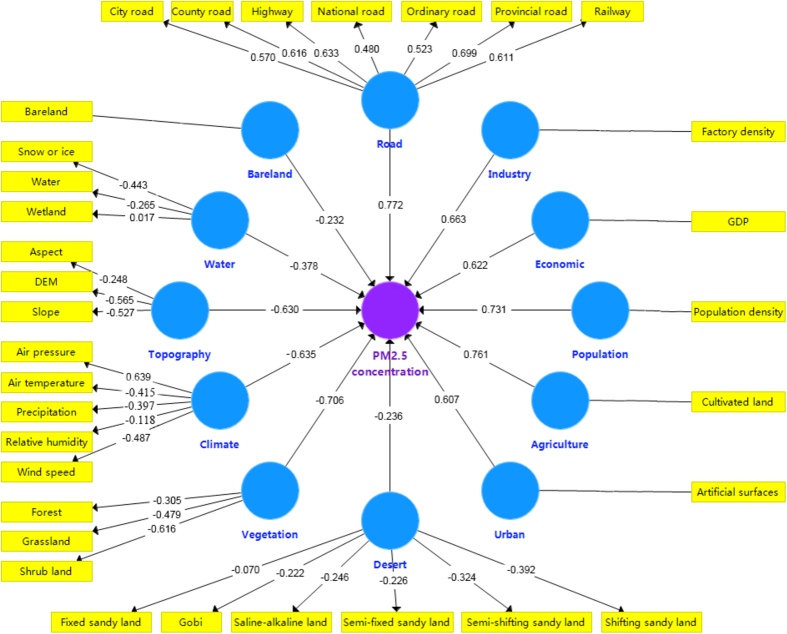
The correlation coefficients between latent geographic factors and PM_2.5_ concentrations. The latent geographic factors under the blue circle, including “Road”, “Industry”, “Economic”, “Population”, “Agriculture”, “Urban”, “Desert”, “Vegetation”, “Climate”, “Topography”, “Water”, “Bareland”, were extracted from the variables in the yellow rectangle. For instance, “Climate” was based on “Air pressure”, “Air temperature”, “Precipitation”, “Relative humidity”, “wind speed”. For factors that contain multiple variables, the coefficients between blue circle and yellow rectangle directly are the contribution of the variables to PM_2.5_. Similarly, the coefficients between blue and purple circle are the contribution of the factors to PM_2.5_. For univariate factors, the coefficients between blue and purple circle are also the contribution of the variables to PM_2.5_.

With the help of ordinary least squares (OLS), multivariate correlation analysis was carried out between latent geographic factors and PM_2.5_ concentrations to identify the decisive factors of PM_2.5_ concentrations before GWR. Figure 2 showed all the correlation coefficients between latent geographic factors and PM_2.5_ concentrations. Overall, most of the correlations are significant in 343 cities except for the factors of bareland and desert.

From Fig. 2, there is a significantly positive correlation between road, agriculture, population, industry, economic, urban and PM_2.5_ concentrations. It implies that PM_2.5_ originates primarily from motor vehicle emissions (diesel and gasoline exhaust), dust (road dust, surface dust, building construction dust), industrial and combustion sources including coal and biomass combustion (e.g. straw, bark residuals, sawdust and shavings)^15^,^16^,^28^,^31^. In particular, traffic (47.9%) and combustion (29.7%) aerosol were two conclusive factors of PM_2.5_ concentrations^22^. This is consistent with the results of our study that road and agriculture have the strongest relationships with PM_2.5_ concentrations.

A significantly negative correlation was found between vegetation and PM_2.5_ concentrations. Previous studies have shown that vegetation can mitigate particulate air pollution through a number of mechanisms, such as intercepting and accumulating atmospheric particles through leaf pubescence and stomata^18^,^19^,^20^. The maximum effect on PM_2.5_ concentrations is the shrub land, followed by the grass land and forest. This is due to the fact that different species have different properties, such as leaf size, stomata, vegetation structure and leaf microstructure which will affect the capture efficiency^21^, whilst forest may influence the microclimate, e.g. blocking effective ventilation and thus lead to higher local PM_2.5_ concentrations^22^.

The results also indicated that there is a significantly negative correlation among all variables of topography and PM_2.5_ concentrations. The decreasing order of ranking is that DEM (altitude), slope and aspect. This may be the reason why PM_2.5_ concentrations of the Sichuan Basin are much higher than that of its circumjacent regions.

Meanwhile, the correlations between all variables of climate and PM_2.5_ concentrations were negative except air pressure. That is to say, an increase (decrease) of each meteorological factor except air pressure will result in the decrease (increase) of PM_2.5_ concentrations. Specifically, there was a moderate negative relation between air temperature and PM_2.5_ concentrations, with a correlation coefficient of −0.415. This is because when temperature rises, air convection becomes quick and frequent, which leads to the diffusion and dilution of PM_2.5_, decreasing PM_2.5_ concentrations, and vice versa. There was a moderate negative correlation between wind speed and PM_2.5_ concentrations, with a correlation coefficient of −0.487 and higher wind speed is conducive to the diffusion of PM_2.5_, which results in lower concentrations of PM_2.5_. A weak negative correlation was found between relative humidity and PM_2.5_ concentrations because when air humidity increases, particles will adsorb moisture, occur condensation, and finally fall in the form of precipitation that results in lower concentrations of PM_2.5_^23^. The correlation coefficients between precipitation and PM_2.5_ concentrations was −0.397 and there are two main impacts of precipitation on PM_2.5_. One is that the adsorption and collision of raindrops upon PM_2.5_ result in the wet sedimentation of PM_2.5_. The other one is that after rainy weather PM_2.5_ concentrations significantly decrease due to the notable reducing of dust and fugitive dust which previously suspended in the atmosphere. PM_2.5_ concentrations display a strong positive correlation with air pressure, with significant correlation coefficients of 0.639. This is due to the fact that when there was high pressure, the down draft hinders the upward movement of PM_2.5_, causing an accumulation of particles^24^.

However, there are weak negative correlations among all variables of desert and bare land. The Variance Inflation Factor (VIF) was used to detect whether collinearity problems existed among the variables by OLS. It turned out that the VIF values of most variables are less than 7.5 except water. In conclusion, socioeconomic factors of road, agriculture, population, industry, economic, urban and natural geographic factors of topography, climate and vegetation were chosen as independent variables of the GWR model.

A higher R^2^ value means that the explanatory variables explain more variance in PM_2.5_ concentrations. From the report of the GWR model, the R^2^ value of GWR was 0.9633, whereas it was 0.6788 of OLS, indicating that the global OLS model only can explain 67.88% of the variance in PM_2.5_ concentrations, but the GWR method had a significant improvement. In terms of the analysis of variance (ANOVA), the model fit at a significant level (F = 13.97, *p* < 0.01), indicating that the GWR model outperformed the OLS model. Moreover, the corrected Akaike information criterion (AICc)^32^ value of GWR (720.18) was much lower than that of the OLS (1182.77). In other words, the performance of GWR model was much better than that of the global OLS model. Through golden-section search^33^,^34^, a bandwidth size of 60 was selected as an appropriate value for the GWR model, and it means that 60 samples were provided for each local estimation within the adaptive bi-square kernel.

**Figure 3:**
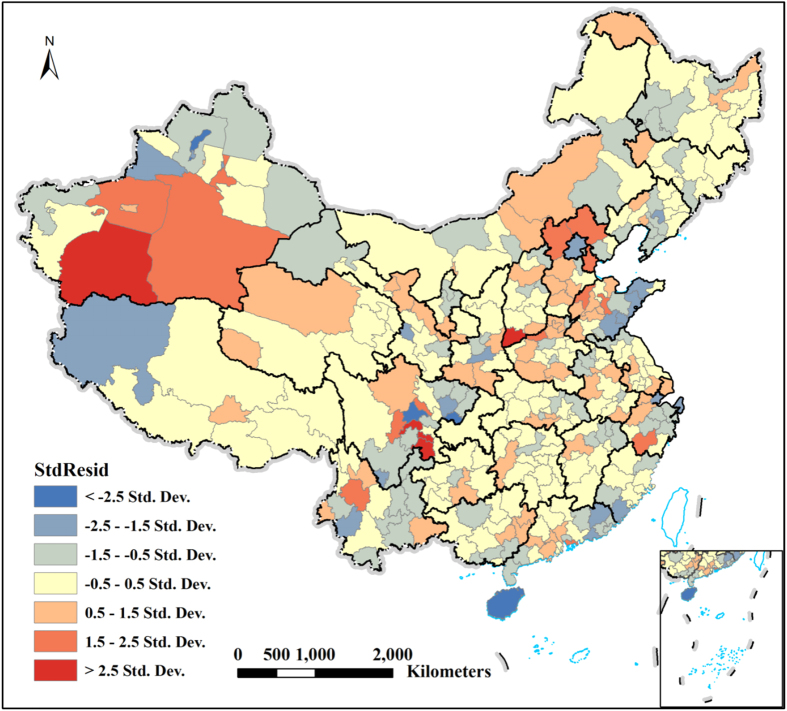
Spatial heterogeneity of standardized residuals for PM_2.5_ concentrations derived from the GWR model. All the maps were generated in ArcGIS10.2, URL: http://www.esrichina-bj.cn/softwareproduct/ArcGIS/.

Figure 3 shows the spatial heterogeneity of standardized residuals for PM_2.5_ concentrations derived from the GWR model and spatial autocorrelation analysis. As it shown, the regions with studentized residual (StdResid) value between −2 and 2 account for 93.58% of the whole Mainland China, which indicates that the relations between each of the nine factors and PM_2.5_ are stable. However, few unusually high (red areas) or low (blue areas) residuals can be observed. Red areas are under predictions in which the actual PM_2.5_ concentrations are higher than the model fitted value. Blue areas are over predictions in which the actual PM_2.5_ concentrations are lower than fitted value. Regions that have a notable under-prediction of PM_2.5_ concentrations need further examination to detect the possible explanations. For instance, those regions of the Tarim Basin in some desert areas, e.g. Hotan prefecture and Bayingolin Mongol Autonomous prefecture have highest residuals (StdResid >2). This is because that the higher concentrations of PM_2.5_ in the desert regions are mainly connected with sand and dust weather phenomena^11^. The Yuncheng city of southern Shanxi province and Tianjin city have much higher residuals because they are rich in marine salt and coal mine. The eastern Sichuan basin, e.g. Deyang, Zigong and Yibin city has high residuals because of its unique geographical climate conditions^35^. Shenzhen city of southern Guangdong province also has much higher residuals because of the influence of coal used as fuel in this area for industrial plants^27^,^36^. Spatial autocorrelation among residuals of GWR was detected by Moran’s I test. Moran’s I for residuals is 0.025 and is not significant at a 0.01 level (Z-score = 1.550, *p* = 0.1212), indicating that the pattern of the residuals does not appear to be significantly different than random. It also suggests that the GWR model does not miss any key explanatory variables and it is properly specified.

**Figure 4:**
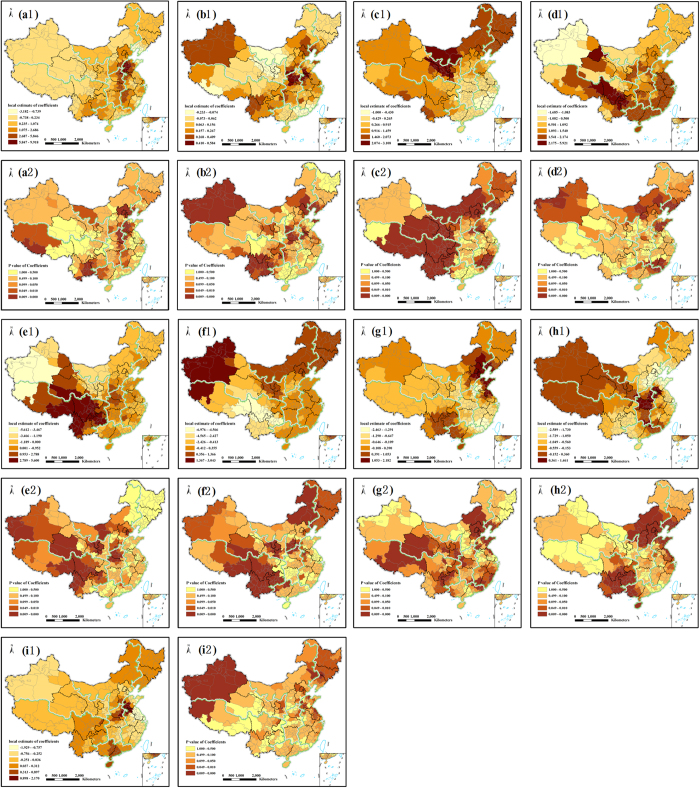
Spatial distribution of local estimates (**a1**–**i1**) and p values (**a2**–**i2**) of nine factors’ coefficients derived from the GWR model. Local estimates of (**a1**) Road, (**b1**) Agriculture, (**c1**) Population, (**d1**) Industry, (**e1**) Economic, (**f1**) Urban, (**g1**) Topography, (**h1**) Climate, (**i1**) Vegetation, *p* values of (**a2**) Road, (**b2**) Agriculture, (**c2**) Population, (**d2**) Industry, (**e2**) Economic, (**f2**) Urban, (**g2**) Topography, (**h2**) Climate, (**i2**) Vegetation. All the maps were generated in ArcGIS10.2, URL: http://www.esrichina-bj.cn/softwareproduct/ArcGIS/.

The GWR model also indicates that the effects of independent variables on PM_2.5_ concentrations vary across space. Figure 4 shows the classification maps for the local estimates and p values of nine factors’ regression coefficients. These results clearly illustrate the existence of an unstable local spatial dependence between PM_2.5_ concentrations and its nine latent geographical factors. In general, it is evident that all coefficients resulting from global models are significant. Such inference is also strong for local coefficients in terms of sign and magnitude.

Specifically, although local parameter estimates for “Road” are positive in most areas of Mainland China, the intensity of the relationships is not constant (Fig. 4(a1)). The positive and strong relationships are found in the northern regions of East China, southern districts of Northwest, North and East China, and a distinct region located in South China (Fig. 4(a2)). In such areas, the effect of “Road” upon PM_2.5_ is relatively higher than in other areas, which indicates very efficient strategies to abate the PM_2.5_ concentrations. The map is produced for “Agriculture” (Fig. 4(b1)) illustrates that there are strong positive trends in the central parts towards the northeast and southwest, while weak negative relationships in the North and South China. Meanwhile, the regression coefficients of “Agriculture” were primarily significant, except in South China, northern areas of the Northeast and East China, and central districts of Southwest China (Fig. 4(b2)). In Southwest China, North China, Northeast China and southern regions of Northwest China, the regression coefficients of “Population” were notable positive (Fig. 4(c1).

The direct effect of “Industry” has been illustrated with greater magnitude in the central areas of Southwest and Northwest China, whole of South China, most areas of East China, and distinct areas of North and Central China (Fig. 4(d1)). And the regression coefficients were primarily significant in the northern regions of the Northwest, North, South and East China, southern areas of the Northeast and East China (Fig. 4(d2)). In most areas, the local coefficient estimates of “Economic” were positive. Comparatively, it is noteworthy that negative estimates were found in some areas. These results indicated that an inverted-U shaped Environmental Kuznets Curve (EKC)^27^ relationship between GDP and PM_2.5_ concentrations indeed exists. Most estimates were significant except in Northeast China, South China, central areas of East China and eastern regions of North China (Fig. 4(e1). The regression coefficient estimates of “Urban” were primarily positive in the west areas of Northwest and Southwest China, Northeast and North China, and the estimates were highly significant in Northeast China, northeast and south of North China, south and central regions of Southwest China and most areas of Northwest China (Fig. 4(f1).

“Topography” had negative effects on “PM_2.5_ Concentrations” except in the southern regions of Southwest and Northeast China, western areas of South China, and eastern districts of East and North China. And their relationships were significant in most areas of Northwest and Southwest China, north and south areas of East China, and the distinct regions of North and Central China (Fig. 4(g1). Estimated local coefficients of “Climate” were for the most part negative over the study area which echoes its direct effect on “PM_2.5_ Concentrations” (Fig. 4(h1)). The stronger effects were found in the central districts of North China, toward eastern expansion. And in most regions of North China, west of South China, east of East China, and south of the Northeast and Southwest China, the estimates were significant (Fig. 4(h2)). In Fig. 4(i1), the direct effect of “Vegetation” has been illustrated with greater magnitude in the southeast areas of Central China, eastern and western regions of Northwest China, some specific areas of North China, and most areas of East China. And its regression coefficients in Northeast China, western regions of Northwest China and Southwest China and some districts of East China and North China were significant (Fig. 4(i2)).

It is expected that the variables such as road, agriculture, industry show direct positive effects on PM_2.5_ concentrations, the variables like population, urban with negative signs are also detectable. Moreover, the variables of topography, climate and vegetation are the opposite. One reason for such counterintuitive signs as expressed by Chow *et al*.^37^ is that the collinearity among some independent variables or the collinearity in estimated local coefficients. It is argued that the local coefficients could be correlated even when there is no collinearity among independent variables^38^. Another reason is that some variables might be less significant at certain locations than others or are completely insignificant, which is due to the method employed in estimating the standard deviation in GWR models^39^.

### Measures and suggestions for controlling PM_2.5_ concentrations

Given the fact that PM_2.5_ pollutions in Mainland China is more and more severe, it is urgent to take quick measures. For example, actions to strictly forbid straw-burning, install desulphurization and dust removal device for coal-fired power plant, close heavily polluting industry, transfer other large industries elsewhere and replace coal with cleaner renewable energy sources, e.g., solar energy, hydrogen fuel, geothermal. Another effective measure to reduce PM_2.5_ emissions is reducing the use of motor vehicles. Some megacities (e.g., Beijing and Shanghai) have limited the vehicles usage through odd-and-even license plate rule and strict control of high displacement automobiles usage. Such measures could be effective in the short term, but will give considerably rise to the government cost and difficult to control in the near future when the pollution became more intense and integrated with regional economic issues^28^.

To fundamentally mitigate PM_2.5_ concentrations, the following suggestions are proposed based on our study and the opinions of other investigations. Firstly, the improvement of fuel quality and the implementation of a more stringent vehicle emission standard are the most effective means of reducing PM_2.5_ emissions. Secondly, formulating strict monitoring system, immediately setting out to a nationwide monitoring network, making contingency plans for heavily polluted days and developing mitigation targets are the important means of governing PM_2.5_. Regional central heating in winter is a substantial measure of the prevention and control of urban PM_2.5_. Thirdly, the environmental protection policy should be prevention-driven rather than problem-driven. It is also important to emphasize that the impacts of each latent factor on PM_2.5_ in different regions are different. Thus, measures and strategies for controlling PM_2.5_ should be an integration of unified planning with the principle of adaptation to local conditions, e.g. the design of vegetation configuration based on local conditions to achieve the goal of increasing vegetation coverage. Furthermore, because of limited communication and lack of a negotiation process, we did not see significant trust building either among the public or between the public and the government, which could have been a major benefit of public participation^40^. Hence the last and the most important suggestions are raising public awareness of PM_2.5_ perniciousness and environmental conservation and strengthening the involvement of the public and government.

### Limitations and future research directions

The models and datasets used in this study reflected an integration of multiple scales, which would inevitably generate uncertainties in spatial statistics. Gridded population and GDP datasets are secondary derived data, and to some extent will introduce new uncertainty. Although there were no collinearity problems existed among the decisive factors, the information redundancy still existed. It affects the performance of the model and even produces counterintuitive signs.

As we all know, the correlations between PM_2.5_ and latent geographic factors that varied at different spatial and temporal scales; therefore, when analyzing their causal relationships, the scale effect should be considered to successfully uncover the spatial and temporal characteristics of PM_2.5_ concentrations. Furthermore, in future studies, it will be necessary to distinguish the influencing ranges of different latent geographic factors and to make certain the inconsistency of their reacting ranges.

## Conclusions

In order to arouse the attention of researchers to investigate the causes of severe PM_2.5_ mass frequently in China nowadays from the macroscopic perspective, this paper analyzed the characteristics of spatiotemporal variations of PM_2.5_ concentrations in Mainland China during 1998–2012. This study was an initial attempt to explore the influencing factors, including natural geographical and socioeconomic factors of PM_2.5_ concentrations in 343 cities across Mainland China and dynamically evaluated the potential health risks of PM_2.5_ in 2000, 2005 and 2010. The following conclusions were drawn from this research:

(1) PM_2.5_ concentrations increased over most of Mainland China during the period 1998–2012. The proportion of area with low PM_2.5_ concentrations less than the WHO AQG of 10 μg/m^3^ declined significantly from 32.92% to 25.67%, while the proportion of area under high range concentrations greater than the WHO Interim Target-1 concentration of 35 μg/m^3^ increased significantly from 23.08% to 29.89%. The concentrations were particularly high in Central, North, Southwest and East China, and the most severe were in northern Henan, southern Hebei and Shanxi, central Shaanxi, eastern Sichuan and western Shandong.

(2) A significantly strong positive correlation was found between all variables of socioeconomic factors (road, agriculture, population, industry, economic, urban) and PM_2.5_ concentrations, while a significantly strong negative correlation was found between almost all variables of natural geographical factors (vegetation, topography, climate) and PM_2.5_ concentrations. Moreover, Moran’s I for its residuals was 0.025 and was not significant at a 0.01 level (Z-score = 1.550, *p* = 0.1212), indicating that the GWR model didn’t miss any key explanatory variables and was properly specified. It is also necessary to emphasize that the effects of each latent factor on PM_2.5_ in various regions are different. Therefore, regional measures and strategies for controlling PM_2.5_ should be integration of unified planning with the principle of adaptation to local conditions.

## Methods

**Figure 5:**
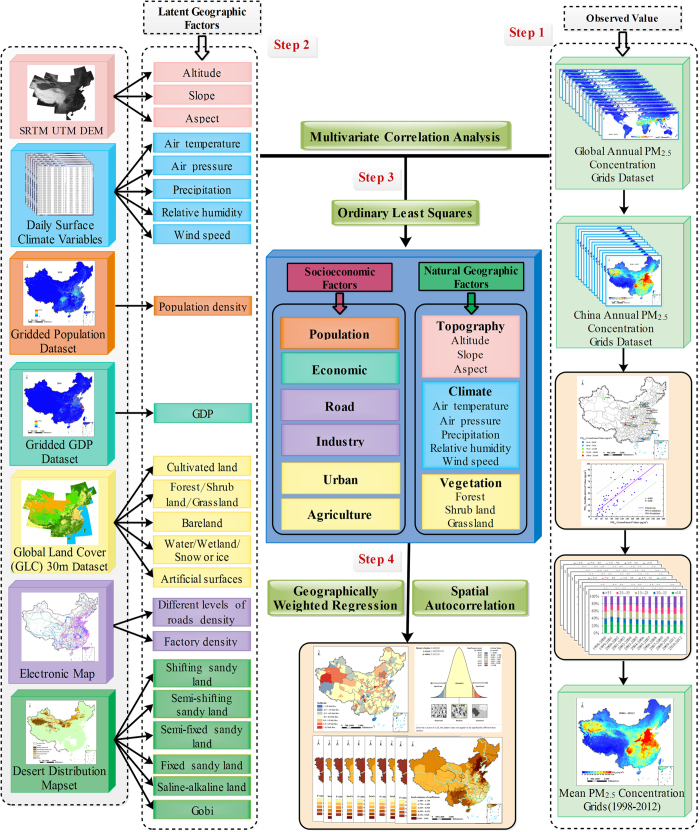
The technical flowchart of this study. All the maps were generated in ArcGIS10.2, URL: http://www.esrichina-bj.cn/softwareproduct/ArcGIS/.

The technical flowchart of this study is shown in Fig. 5, mainly includes the following steps:

Step 1: Validate the accuracy of the annual PM_2.5_ concentrations grids dataset in Mainland China based on related literatures and evaluate the spatial and temporal characteristics of PM_2.5_ exposures from 1998 to 2012.

Step 2: Download data and extract latent geographic factors from these datasets.

Step 3: Analyze and compare the impact of each latent geographic factor on PM_2.5_ concentrations through multivariate correlation analysis. Moreover, calculate the VIF by OLS to detect whether collinearity problems existed among the factors. The goal of this step is to determine the decisive factors on PM_2.5_ concentrations.

Step 4: Apply the GWR method to explore spatial non-stationarity and varying relationships between PM_2.5_ concentrations and decisive factors. Moreover, spatial autocorrelation analysis was used to detect the performance of the GWR.

In brief, conventional statistical analysis and GWR model were adopted in this study.

### Conventional statistical analysis

In order to make the analysis straightforward, annual average PM_2.5_ concentrations were categorized sequentially into six grades (Grade 1: <10, Grade 2: 10–15, Grade 3: 15–25, Grade 4: 25–35, Grade 5: 35–100, Grade 6: >100 μg/m^3^) according to WHO’s air quality guidelines^2^ and the latest version of China’s ambient air quality standard (GB 3095–2012)^41^. WHO’s air quality guidelines have four standards, including one air quality guideline (AQG:10 μg/m^3^) and three interim targets. As for the interim targets, Interim Target-1 (IT-1:35 μg/m^3^) was in line with the annual average secondary standard of PM_2.5_ concentrations of China’s ambient air quality standard; Interim Target-2 (IT-2:25 μg/m^3^) and Interim Target-3 (IT-3:15 μg/m^3^) was consistent with the annual average primary standard of PM_2.5_ concentrations of China’s ambient air quality standard. The characteristics of the variations in the seven regions were analyzed to quantify the degree of spatial variation across Mainland China. To objectively analyze the influencing factors of PM_2.5_ concentrations in Mainland China as well as to reduce the influence of data from different years, mean PM_2.5_ concentrations were calculated at city level using the PM_2.5_ concentrations dataset and city boundary layer derived from electronic map. Similarly, values of each latent geographic factors (e.g. population density, road density, slope, wind speed, forest cover ratio, etc.) of 343 cities were also summarized.

### Geographically weighted regression

Geographically weighted regression (GWR) was adopted to explore the local spatial heterogeneity of the causal relationships between PM_2.5_ concentrations and geographic factors. It is a powerful technique to examine geographically non-stationarity and varying relationships between dependent/response variable Y and a set of independent/explanatory variables *X*_*j*_ (*j* = *1, 2, …, m*) at regional scale^42^. Through adding the geographical location information into the conventional regression process, the GWR attempts to show how the relationship between the dependent variable and the independent variables varies over the entire space. Also, it is described by the equation:


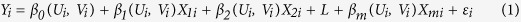


where (*U*_*i*_, *V*_*i*_) and *ε*_*i*_ are respectively space coordinate and regression residual of the *i*th location. And unlike conventional global regression, the coefficients *β*_*j*_(*U*_*i*_, *V*_*i*_) (*j* = *1, 2*, …, *m*) are varying conditionals on the location.

In GWR model, the regression coefficients show the local spatial variation, and the standard errors of the coefficients illustrate the reliability of the estimated coefficients^43^. Considering the samples are not regularly spaced in our study, GWR v4.0^33^,^34^ with the adaptive bandwidth and bi-square kernel was implemented to build the model. Meanwhile, golden-section search which can efficiently identify the optimal bandwidth size in most cases was used in this research. Furthermore, the AICc was extensively adopted to compare the global OLS model with a local GWR model.

## Data and Study Area

### Study area

Cities, including county-level, prefectural-level cities and municipalities are the basic administrative units which can be used to reveal Chinese Mainland’s natural geographic features and socioeconomic condition, as well as its air pollution. Additionally, natural geographic factors and socioeconomic factors match well with the PM_2.5_ concentrations distribution at the city level. According to the above mentioned analysis, 343 cities were used as the basic study unit to explore the influencing factors of PM_2.5_ in Mainland China. In order to better and more easily elaborate the results, Mainland China was divided into seven geographical subareas: Northeast China (NEC), North China (NC), East China (EC), Central China (CC), South China (SC), Southwest China (SWC), and Northwest China (NWC) (Table 1).

Multisource data used in this study is listed in Table 2, which is classified into two broad categories: 1) PM_2.5_ data used as dependent/response variable Y in GWR model; 2) natural geographical and socioeconomic data, such as DEM, population, GDP and so on used as independent/explanatory variables X_j_ (*j* = *1, 2*, …, *m*).

### PM_2.5_ data

There is a lack of publically available global remote sensing data related to PM_2.5_, until van Donkelaar *et al*.^4^,^5^,^6^ used the GEOS-Chem global chemical transport model (http://geos-chem.org/) to successfully map global ground-level PM_2.5_ concentrations. They are based on total column aerosol optical depth (AOD) from a combination of MODIS (Moderate Resolution Imaging SpectroRadiometer), MISR (Multi-angle Imaging SpectroRadiometer) and SeaWIFS (Sea-Viewing Wide Field-of-View Sensor) AOD satellite instruments and coincident aerosol vertical profiles. The global annual PM_2.5_ concentrations grids dataset represents a series of three-year running mean grids (1998–2012) of fine particulate matter (solid particles and liquid droplets) that provides the highest accuracy, largest coverage (from 70°N to 55°S), longest temporal range and highest resolution (6 arc-minutes, 0.1 degree or approximately 10 km at the equator). It has been effectively applied on a national and regional scale^26^,^28^,^44^. These data is derived from Socioeconomic Data and Applications Center (sedac)—Hosted by the Center for International Earth Science Information Network (CIESIN) at Columbia University (http://sedac.ciesin.columbia.edu/). A subset of the global PM_2.5_ concentrations grids dataset (1998–2012) covering the Mainland China was used in this research. Furthermore, there exist data voids in the original PM_2.5_ dataset due to snow-covered mountains, perennial cloud and sensor malfunctions, in particular for the Tibetan Plateau. With the purpose of alleviating this problem, the spline interpolation method^45^ was utilized to make up data voids.

Although van Donkelaar *et al*. has validated the accuracy of the global annual PM_2.5_ concentrations grids dataset based on the agreement between satellite-derived estimates and ground-based measurements^5^,^6^. However, considering that the relationship between AOD-PM_2.5_ can differ by space and countries, it was still necessary to evaluate the reliability of the dataset for the specific area of Mainland China. Because China has not built national PM_2.5_ network monitoring sites until the end of 2012, continuous observation data with which to validate satellite-derived air quality data was not available. Therefore, we collected Chinese ground-based PM_2.5_ measurements of different sites at different times from relevant published literature. Consequently, as Supplementary Table S1 shown, 61 sample points with site location, geocoordinates, ground-based PM_2.5_ values, and sampling periods were extracted from 34 relevant studies.

**Figure 6:**
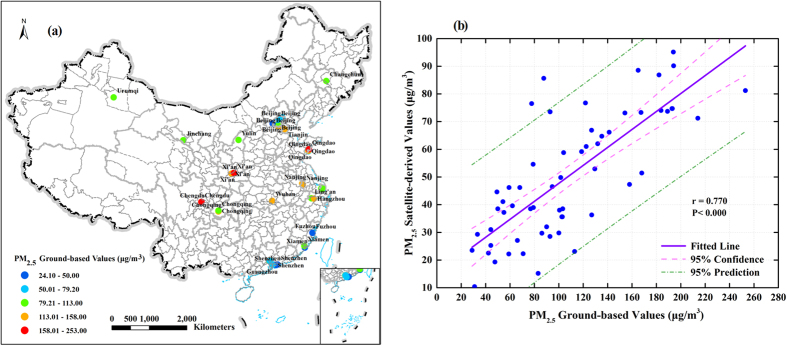
(**a**) The ground-based PM_2.5_ measurements collected from the literature for locations in Mainland China and (**b**) linear correlation of the two datasets (PM_2.5_ ground-based values and satellite-derived values). The left map was generated in ArcGIS10.2, URL: http://www.esrichina-bj.cn/softwareproduct/ArcGIS/.

The ground measured PM_2.5_ concentrations and spatial distribution of these sample points are shown in Fig. 6(a). Furthermore, according to the location and period of the sample points, the corresponding satellite-derived values of PM_2.5_ concentration data were calculated. Also, the linear correlation between “PM_2.5_ Ground-based Values” and “PM_2.5_ Satellite-derived Values” is shown in Fig. 6(b). A significant overall agreement is found (r = 0.770), which indicated that the satellite-derived PM_2.5_ concentration data were reliable. The agreement was higher than that for Europe (r = 0.730) and North America (r = 0.760)^6^. However, the satellite-derived values tend to be lower than the ground-based values, and this was consistent with results of Europe and North America^6^. Some of this underestimate may arise from the different PM_2.5_ measurement methods which were used in remote sensing and site monitoring. While others might be due to that the time spans of the sample points and remote sensing were inconsistent, e.g., some sample points were monitored only in a few days or months of one year, while remote sensing monitored yearly.

### Natural geographical and socioeconomic data

1. DEM data: The SRTM UTM DEM was obtained from the processing of a void-filled version of the SRTM3 dataset (SRTM3 V4.1). The SRTM3 V4.1 was offered by the International Center for Tropical Agriculture (CIAT) and its data gaps were filled with different interpolation algorithms^46^ and SRTM DEM. The SRTM DEM was acquired by radar interferometry (InSAR) during about ten days in February 2000, when a Space Shuttle mapped the Earth surface between 60°N and 56°S with C-band radar. Based on these data and other products, a covering more than 80% of earth’s land surface DEM with three arc-seconds resolution (~90 m, SRTM3) was derived and distributed for free in 2003. This data has been validated through comparison with ground control points. SRTM UTM DEM is downloaded from International Scientific & Technical Data Mirror Site, Computer Network Information Center, and Chinese Academy of Sciences (http://www.gscloud.cn).
2. Meteorological site data: Meteorological site data are derived from the “Daily Surface Climate Variables of China” catalog (SURF_CLI_CHN_MUL_DAY_V3.0 on June-25-2014), which is released by the Climatic Data Center, National Meteorological Information Center, China Meteorological Administration and China Meteorological Data Sharing Service System (http://cdc.cma.gov.cn/home.do). The dataset starts on 1 January, 1951, and keeps running till now, with a total of 824 fundamental stations (The number of stations does vary in a different year, but has remained stable since 1980) throughout Mainland China. It consists of over 22 climate variables, although we focus on daily average air temperature (TEM), average air pressure (PRS), average relative humidity (RHU), average wind speed (WIN) and total precipitation (PRE) in this study. The raster/grid maps with 1 km resolution of the annual mean air temperature, air pressure, wind speed, rainfall and relative humidity are generated using thin plate spline spatial interpolation method^47^. The spline surfaces are fitted as functions of latitude, longitude, and elevation. The same elevation data used is SRTM UTM DEM 90 m data. It is noted that the elevation is a co-predictor, and thus a topographic correction for the gridded data is calculated during the interpolation.
3. GDP and population data: GDP and population are common indicators of socioeconomic development. The gridded GDP (2010) and population datasets (2010) in Mainland China were all provided by the National Data Sharing Infrastructure of System Science (http://www2.geodata.cn/index.html). Respectively according to the relationship between demographic data, GDP data and land use types, these gridded datasets with a spatial resolution of 1 km were transformed from statistical yearbook^48^.
4. Land cover data: Global Land Cover 30 m dataset (GLC30) was downloaded from Global Land Cover Information Service System (http://www.globallandcover.com/GLC30Download/index.aspx). This kind of land cover data was derived by using multisource data, including Landsat TM, ETM+, Environmental Disaster Alleviation Satellite (HJ-1) multispectral images. The dataset has a grid cell resolution of 30 m, covers the global land surface from 80°N to 80°S, and consists of ten land cover types: cultivated land, forest, grassland, shrubland, wetland, water bodies, tundra, artificial surfaces, bareland, permanent snow and ice.
5. Desert distribution mapset and electronic map: As with the GDP and population datasets, Desert distribution Mapset was also derived from the National Data Sharing Infrastructure of System Science (http://www2.geodata.cn/index.html). This mapset covers most area of Mainland China excluding Hunan, Yunnan province, Shanghai and Chongqing municipality. Desert was divided into six types: shifting sandy land, semi-shifting sandy land, fixed sandy land, semi-fixed sandy land, saline-alkaline land and gobi.

Administrative boundary, factory and road density of Mainland China used in this work were extracted from an electronic map in 2010. All datasets were rectified to the Universal Transverse Mercator (UTM) projection system (datum WGS 1984, zone 48), and integrated into a geodatabase.

## Additional Information

**How to cite this article**: Luo, J. *et al*. Spatiotemporal Pattern of PM_2.5_ Concentrations in Mainland China and Analysis of Its Influencing Factors using Geographically Weighted Regression. *Sci. Rep.* **7**, 40607; doi: 10.1038/srep40607 (2017).

**Publisher's note:** Springer Nature remains neutral with regard to jurisdictional claims in published maps and institutional affiliations.

## Supplementary Material

Supplementary Information

## Figures and Tables

**Table 1:** Seven geographical subareas of Mainland China.

Geographical subarea	Provinces and municipalities
Northeast China	Liaoning, Jilin, and Heilongjiang
North China	Beijing, Tianjin, Hebei, Shanxi and Inner Mongolia
East China	Jiangsu, Zhejiang, Shanghai, Anhui, Fujian, Shandong and Jiangxi
Central China	Hunan, Hubei and Henan
South China	Guangxi, Hainan and Guangdong
Southwest China	Sichuan, Yunnan, Guizhou, Chongqing and Tibet
Northwest China	Gansu, Qinghai, Ningxia, Shaanxi and Xinjiang

**Table 2:** Basic information of the eight datasets used in this study.

Data	Sources	Year	Resolution or scale
Global Annual PM_2.5_ Grids Datasets	http://sedac.ciesin.columbia.edu/	1998–2012	10000 m
SRTM UTM DEM	http://www.gscloud.cn	2003	90 m
Daily Surface Climate Variables of China	http://cdc.cma.gov.cn/home.do	2010	1000 m
Gridded GDP Dataset	http://www2.geodata.cn/index.html	2010	1000 m
Gridded Population Dataset	http://www2.geodata.cn/index.html	2010	1000 m
Global Land Cover 30 m Dataset	http://www.globallandcover.com/GLC30Download/index.aspx	2004–2010	30 m
Desert Distribution Mapset	http://www2.geodata.cn/index.html	2000	1:100000
Electronic Map		2010	1:10000
