# Supplementary material for: Spatiotemporal Pattern of PM2.5 Concentrations in Mainland China and Analysis of Its Influencing Factors using Geographically Weighted Regression
Source: Sci Rep. 2017 Jan 12;7:40607. doi: 10.1038/srep40607 (PMC5228184; doi:10.1038/srep40607)
Supplement: Supplementary Information [file srep40607-s1.pdf]

Spatiotemporal Pattern of $PM_{2.5}$ Concentrations in Mainland China and Analysis of Its Influencing Factors using Geographically Weighted Regression

Jieqiong Luo[1,2,3], Peijun Du[1,2,3*], Alim Samat[4], Junshi Xia[5], Meiqin Che[1,2,3], Zhaohui Xue[6]

1. Key Laboratory for Satellite Mapping Technology and Applications of State Administration of Surveying, Mapping and Geoinformation of China, Nanjing University, Nanjing 210023, China;
2. Jiangsu Provincial Key Laboratory of Geographic Information Science and Technology, Nanjing University, Nanjing 210023, China
3. Jiangsu Center for Collaborative Innovation in Geographical Information Resource Development and Application
4. State Key Laboratory of Desert and Oasis Ecology, Xinjiang Institute of Ecology and Geography, Chinese Academy of Sciences, Urumqi 830011, China
5. Research Center for Advanced Science and Technology, The University of Tokyo 4-6-1 Komaba, Meguro-ku, Tokyo 153-8904, Japan
6. School of Earth Sciences and Engineering, Hohai University, Nanjing 211100, China

*Corresponding author Email: dupjrs@126.com

16

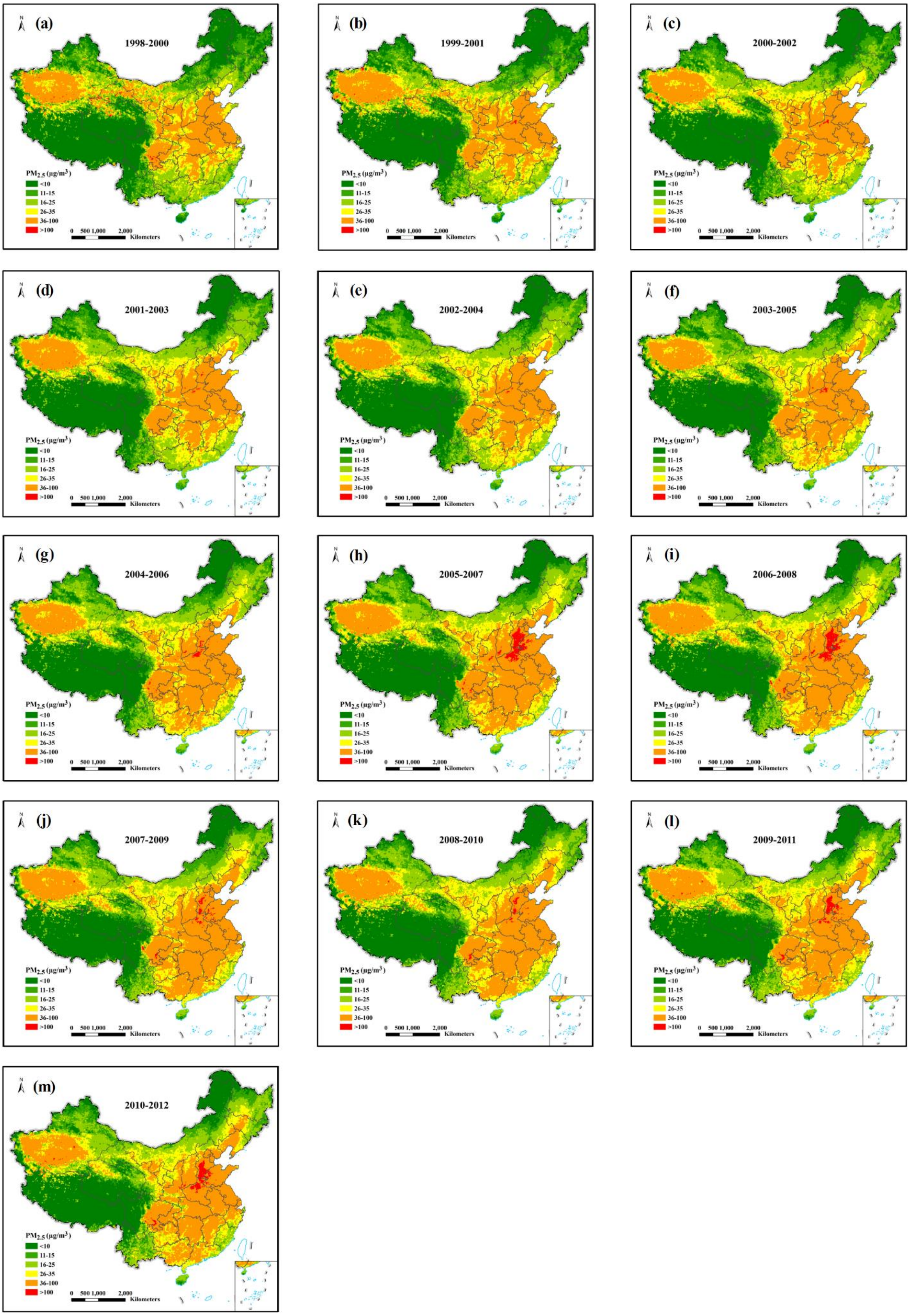

Figure S1. Spatial distribution of annual average $PM_{2.5}$ concentrations across Mainland China during 1998-2012. (a) 1998-2000, (b) 1999-2001, (c) 2000-2002, (d) 2001-2003, (e)2002-2004, (f) 2003-2005, (g) 2004-2006, (h) 2005-2007, (i) 2006-2008, (j) 2007-2009, (k) 2008-2010, (l)2009-2011, (m) 2010-2012. All the maps were generated in ArcGIS10.2, URL: http://www.esrichina-bj.cn/softwareproduct/ArcGIS/

Table S1 **Basic information of 61 sample points which were extracted from 34 relevant studies.**

No	Sites	Longitude	Latitude	Ground-based values (μg/m^3)	Sampling period	Reference
1	Beijing	116.28	39.54	154.26	2001-2003	Wang et al.(2005) [1]
2	Beijing	116.4	39.9	121.85	2003.1,7	Cao et al.(2007) [2]
3	Beijing	116.34	40	77.8	2003	Wang et al.(2010) [3]
4	Beijing	116.3	39.9	87.7	2005-2007	Zhao et al.(2009) [4]
5	Beijing	117.1	40.6	54.2	2005-2007	Zhao et al.(2009) [4]
6	Beijing	115.43	39.97	33.3	2009.9-2011.9	Xin et al.(2014) [5]
7	Beijing	116.3	40.35	59.2	2005.6.20-8.6	Wu and Wang(2007) [6]
8	Beijing	116.3	40.35	68	2005.6.29-8.2	Pathak et al.(2011) [7]
9	Changchun	125.3	43.9	100.05	2003.1,7	Cao et al.(2007) [2]
10	Chengdu	104.02	30.66	165.1	2009.4-2010.1	Tao et al.(2013) [8]
11	Chengdu	104.07	30.68	165.1	2009.4.29-5.17,7.6-8.6,10.26-11.26, 2010.1.1-1.31	Zhang et al.(2013) [9]
12	Chongqing	106.5	29.5	214.05	2003.1,7	Cao et al.(2007) [2]
13	Chongqing	106.48	29.48	131.9	2012.2-12	Ren et al.(2014) [10]
14	Chongqing	106.49	29.59	104	2010.3-2011.7	Zhang et al.(2012a) [11]
15	Fuzhou	119.3	26.11	44.33	2007.4-2008.1	Xu et al.(2012) [12]
16	Fuzhou	119.32	26.08	44.33	2007.4-2008.1	Xu et al.(2012) [12]
17	Guangzhou	113.2	23.1	102.55	2003.1,7	Cao et al.(2007) [2]
18	Guangzhou	113.26	23.13	102.9	2004.10.5-11.5	Andreae et al. (2008) [13]
19	Guangzhou	113.53	22.7	113	2007.10.23-11.24	Ding et al.(2011) [14]
20	Guangzhou	113.35	23.12	76.8	2009.4-2010.1	Tao et al.(2012) [15]
21	Guangzhou	113.53	22.7	70.8	2010.11.02-12.26	Wang et al.(2012) [16]
22	Guangzhou	113.31	23.16	127	2003.7-2004.6	Li et al. (2005) [17]
23	Guangzhou	113.31	23.1	62	2008	Zhu et al.(2013) [18]
24	Guangzhou	113.31	23.1	50	2009	Zhu et al.(2013) [18]
25	Guangzhou	113.31	23.1	55	2010	Zhu et al.(2013) [18]
26	Guangzhou	113.35	23.12	103.3	2010.1.1-31	Tao et al.(2012) [15]
27	Guangzhou	113.35	23.12	79.2	2007.4.1-30	Tao et al.(2009) [19]
28	Guangzhou	113.62	22.6	59	2004.5.15-27	Pathak et al.(2011) [7]
29	Hangzhou	120.1	30.2	129.6	2003.1,7	Cao et al.(2007) [2]
30	Jinchang	101.1	38.3	83.1	2003.1,7	Cao et al.(2007) [2]
31	Ling'an	119.75	30.28	90	1999.10-11	Xu et al.(2002) [20]
32	Nanjing	118.73	32.21	135.33	2002.12-2003.10	Fan et al.(2005) [21]
33	Nanjing	118.79	32.06	122.5	2002.12-2003.10	Fan et al.(2005) [21]
34	Qingdao	120.44	36.11	49.36	1998–2000	Hu et al.(2002) [22]
35	Qingdao	120.3	36	79	2003.1,7	Cao et al.(2007) [2]

36	Qingdao	120.41	36.23	168	2011.10.25-11.3,2012.2.15-22,3.28-29, 5.3-12,8.22-30	Wu et al.(2013) [23]
37	Qingdao	120.31	36.06	158	2011.10.25-11.3,2012.2.15-22,3.28-29, 5.3-12,8.22-30	Wu et al.(2013) [23]
38	Shanghai	121.5	31.2	66.1	1999.5–2000.3	Ye et al.(2003) [24]
39	Shanghai	121.4	31.2	101.65	2003.1,7	Cao et al.(2007) [2]
40	Shanghai	121.5	31.3	94.64	2003.9-2005.1	Wang et al.(2006) [25]
41	Shanghai	121.3	31.3	92.9	2005.10-2006.7	Feng et al.(2009) [26]
42	Shenzhen	113.9	22.58	42.3	2008.12.30-2009.12.25	Yun et al.(2013) [27]
43	Shenzhen	114.12	22.56	31	2008.12.30-2009.12.25	Yun et al.(2013) [27]
44	Shenzhen	114.3	22.6	28.9	2008.12.30-2009.12.25	Yun et al.(2013) [27]
45	Tianjin	117.2	39.1	141.3	2003.1,7	Cao et al.(2007) [2]
46	Urumqi	89.15	42.6	92.8	2011.1-12	Wang et al.(2016) [28]
47	Wuhan	114.2	30.5	118.7	2003.1,7	Cao et al.(2007) [2]
48	Xiamen	118.1	24.4	47.7	2003.1,7	Cao et al.(2007) [2]
49	Xiamen	118.06	24.61	86.16	2009.6-2010.5	Zhang et al.(2012b) [29]
50	Xi'an	108.9	34.2	253	2003.1,7	Cao et al.(2007) [2]
51	Xi'an	108.54	34.16	183.8	2003.9-2007.7	Liu et al.(2009) [30]
52	Xi'an	108.54	34.16	189.1	2003.9-12	Liu et al.(2009) [30]
53	Xi'an	108.54	34.16	167.4	2004	Liu et al.(2009) [30]
54	Xi'an	108.54	34.16	183.8	2005	Liu et al.(2009) [30]
55	Xi'an	108.54	34.16	193.1	2006	Liu et al.(2009) [30]
56	Xi'an	108.88	34.23	182.2	2006.1-2008.12	Cao et al.(2012) [31]
57	Xi'an	108.88	34.23	194.1	2006.3-2007.3	Zhang et al.(2011) [32]
58	Xi'an	108.54	34.16	192.8	2007.1-7	Liu et al.(2009) [30]
59	Xi'an	108.54	34.16	126.85	2008.7.5-2009.8.8	Liu et al.(2013) [33]
60	Xi'an	108.88	34.23	193.9	2012.12.1-31	Zhang et al.(2015) [34]
61	Yulin	109.8	38.3	100.55	2003.1,7	Cao et al.(2007) [2]

References

1. Wang, Y. et al. The ion chemistry and the source of $PM_{2.5}$ aerosol in Beijing. *Atmos. Environ*. **39**, 3771–3784 (2005).
2. Cao, J. et al. Spatial and seasonal distributions of carbonaceous aerosols over China. *J. Geophys. Res*. **112**, 22-11 (2007).
3. Wang, J., Yang, F., Wang, D., & He, K. Characteristics and relationship of aerosol optical thickness and $PM_{2.5}$ concentration over Beijing. *Journal of the Graduate School of the Chinese Academy of Sciences*, **27**, 10–16 (2010) (In Chinese).
4. Zhao, X. et al. Seasonal and diurnal variations of ambient $PM_{2.5}$ concentration in urban and rural environments in Beijing. *Atmos. Environ*. **43**, 2893–2900 (2009).

5. Xin, J. et al. The empirical relationship between the $PM_{2.5}$ concentration and aerosol optical depth over the background of North China from 2009 to 2011. *Atmos. Res*. **138**, 179–188 (2014).
6. Wu,S., Wang,T. On the performance of a semi-continuous $PM_{2.5}$, sulphate and nitrate instrument under high loadings of particulate and sulphur dioxide. *Atmos. Environ*. **41**, 5442-5451 (2007)
7. Pathak R K, Wang T, Ho K F, et al. Characteristics of summertime $PM_{2.5}$ organic and elemental carbon in four major Chinese cities: Implications of high acidity for water-soluble organic carbon (WSOC). *Atmos. Environ*. **45**, 318-325 (2011).
8. Tao, J. et al. Chemical composition of $PM_{2.5}$ at an urban site of Chengdu in southwestern China. *Adv.Atmos.Sci*.**30**, 1070–1084 (2013).
9. Zhang, Z., Tao, J., Xie, S., et al. Seasonal variations and source apportionment of $PM_{2.5}$ at urban area of Chengdu. *Acta Scientiae Circumstantiae*. **33**, 2947-2952 (2013) (In Chinese).
10. Ren, L., Zhou, Z., Zhao, X., et al. Source apportionment of PM_{10} and $PM_{2.5}$ in urban areas of Chongqing. *Res. Environ. Sci*. **27**,1387-1394 (2014) (In Chinese).
11. Zhang, D., Zhai, C., Zhou, Z., et al. Characteristics of water-soluble inorganic ions in different size particles in Chongqing. *Res. Environ. Sci*. **25**, 1099-1106 (2012) (In Chinese).
12. Xu, L. et al. Seasonal variations and chemical compositions of $PM_{2.5}$ aerosol in the urban area of Fuzhou, China. *Atmos. Res*. **104**, 264–272 (2012).
13. Andreae, M. O. et al.Optical properties and chemical composition of the atmospheric aerosol in urban Guangzhou, China. *Atmos. Environ*. **42**, 6335–6350 (2008).
14. Ding, X., Wang, X., & Zheng, M. The influence of temperature and aerosol acidity on biogenic secondary organic aerosol tracers: Observations at a rural site in the central Pearl River Delta region, South China. *Atmos. Environ*. **45**, 1303–1311 (2011).
15. Tao, J. et al. Reconstructed light extinction coefficients using chemical compositions of $PM_{2.5}$ in winter in urban Guangzhou, China. Advances in Atmospheric Sciences. *Adv. Atmos. Sci*. **29**, 359–368 (2012).
16. Wang, X. et al. Aerosol scattering coefficients and major chemical compositions of fine particles observed at a rural site hit the central Pearl River Delta, South China. *J Environ Sci-China*. **24**, 72–77 (2012).
17. Li, L. et al. Variations of PM_{10} and $PM_{2.5}$ levels in street microenvironment in Guangzhou. *Earth Environ*. **33**, 57-60 (2005) (In Chinese).
18. Zhu, Q., Liu, Y., Xu, W., Huang, M. Analysis on the Pollution Characteristics and Influence Factors of $PM_{2.5}$ in Guangzhou. *Environ. Monitor. Chin*. **29**, 15-21 (2013) (In Chinese).
19. Tao, J., Ho, K., Chen, L., Zhu, L., et al. Effect of chemical composition of $PM_{2.5}$ on visibility in Guangzhou,China,2007 spring. *Particuology*. **7**, 68-75 (2009).
20. Xu, J., et al. Measurement of aerosol chemical, physical and radiative properties in the Yangtze delta region of China. *Atmos. Environ*. **36**, 161–173 (2002).
21. Fan J., Zheng Y., Wang Z. Compared analysis of element content in atmospheric $PM_{2.5}$ in Nanjing urban and suburban area. *Chin. Environ. Sci*. **25**, 146-150 (2005) (In Chinese).
22. Hu, M. et al. Seasonal variation of ionic species in fine particles at Qingdao, China. *Atmos. Environ*. **36**, 5853–5859 (2002).
23. Wu, h., Zhang, C., Wang, J., et al. Comparative study on pollution characteristics and source apportionment of PM_{10}

and $PM_{2.5}$ in Qingdao. *Res. Environ. Sci*. **26**, 583-589 (2013) (In Chinese).

24. Ye, B. et al. Concentration and chemical composition of $PM_{2.5}$ in Shanghai for a 1-year period. *Atmos. Environ*. **37**, 499–510 (2003).

25. Wang, Y. et al. The ion chemistry, seasonal cycle, and sources of $PM_{2.5}$ and TSP aerosol in Shanghai. *Atmos. Environ*. **40**, 2935–2952 (2006).

26. Feng, Y. et al. Characteristics of organic and elemental carbon in $PM_{2.5}$ samples in Shanghai, China. *Atmos. Environ*. **92**, 434–442 (2009).

27. Yun, H. et al.Characterising Seasonal Variation and Spatial Distribution of $PM_{2.5}$ Species in Shenzhen. *Environ. Sci.* **34**, 1245-1251 (2013) (In Chinese).

28. Wang,G.et al. Seasonal changes of carbonaceous speeies in $PM_{2.5}$, $PM_{2.5\sim10}$ in Urumqi. *Chin Environ Sci*. **36**, 356-362 (2016) (In Chinese).

29. Zhang, F. et al. Chemical compositions and extinction coefficients of $PM_{2.5}$ in peri-urban of Xiamen, China, during June 2009–May 2010. *Atmos. Res*. **106**, 150–158 (2012)

30. Liu, S., Cao, J., & An, Z. Characterization of ambient fine particles ($PM_{2.5}$) concentration and its influential factors in Xi'an. *Chin. J.Proc*. **S2**, 231–236 (2009) (In Chinese).

31. Cao, J. et al. Impacts of aerosol compositions on visibility impairment in Xi'an, China. *Atmos. Environ*. **59**, 559–566 (2012).

32. Zhang, T.et al. Water-soluble ions in atmospheric aerosols measured in Xi'an, China: Seasonal variations and sources. *Atmos. Res*. **102**, 110–119 (2011).

33. Liu, S., Cao, J., Ho, K., Xu, H. Characteristics of water-soluble organic and inorganic nitrogen in atmospheric fine particles($PM_{2.5}$) from Xi'an. *J. Earth Environ*. **4**, 1272–1279 (2013) (In Chinese).

34. Zhang, Q., Shen, Z., Cao, J., et al. Variations in $PM_{2.5}$, TSP, BC, and trace gases (NO_2, SO_2, and O_3) between haze and non-haze episodes in winter over Xi'an, China. *Atmos. Environ*. **112**, 64-71 (2015).
